# Prevalence of and factors associated with acute withdrawal symptoms after 24 weeks of eszopiclone treatment in patients with chronic insomnia: a prospective, interventional study

**DOI:** 10.1186/s12888-021-03196-0

**Published:** 2021-04-14

**Authors:** Yuichi Inoue, Yoshikazu Takaesu, Michinori Koebis

**Affiliations:** 1 Japan Somnology Center, Institute of Neuropsychiatry, 5-10-10 Yoyogi, Shibuya-ku, Tokyo, 151-0053 Japan; 2 Department of Somnology, Tokyo Medical University, 5-10-10 Yoyogi, Shibuya-ku, Tokyo, 151-0053 Japan; 3 Department of Neuropsychiatry, Graduate School of Medicine, University of the Ryukyus, 207 Uehara, Nishihara, Okinawa 903-0215 Japan; 4 Medical Headquarters, Eisai Co., Ltd., Nishigoken-cho 13-1, Shinjuku-ku, Tokyo, 162-0812 Japan

**Keywords:** Benzodiazepine receptor agonists, Eszopiclone, Withdrawal symptoms, Typical clinical dose, Benzodiazepine dependence self report questionnaire

## Abstract

**Background:**

Although long-term use of benzodiazepines and benzodiazepine receptor agonists (BZDs) has been associated with an increased risk of dependence, the incidence, details of clinical manifestations, and triggering factors of withdrawal symptoms associated with long-term BZD use at common clinical doses remain unclear.

**Methods:**

In a multicenter, open-label study of 123 Japanese patients with insomnia, patients were given a common clinical dose of eszopiclone (2 mg) for 24 weeks, and then treatment was abruptly discontinued. Withdrawal symptoms were evaluated using the Benzodiazepine Hypnotics Withdrawal Symptom Scale (BHWSS). The Insomnia Severity Index (ISI) was used to rate insomnia severity during treatment and 2 weeks after discontinuation. Dependence and poor compliance during treatment without strict medication controls were evaluated with the Benzodiazepine Dependence Self Report Questionnaire short version (Bendep-SRQ SV) subscale sum scores for problematic use, preoccupation, and lack of compliance. Associations between the presence of clinically relevant withdrawal symptoms (BHWSS≥7) and demographic measures, ISI scores at Week 24, and Bendep-SRQ SV subscale sum scores were evaluated by multivariable stepwise logistic regression analyses.

**Results:**

Seventy-six patients completed treatment and 2 weeks of withdrawal; eight (10.5%) had clinically relevant withdrawal symptoms. On multiple logistic regression analysis, Bendep-SRQ SV subscale sum scores were correlated with withdrawal symptoms (odds ratio, 1.650; 95% confidence interval, 1.105–2.464; *p* = 0.014). Exacerbation of post-discontinuation insomnia was not significantly different between patients who showed clinically relevant withdrawal symptoms and those who did not (*p* = 0.245).

**Conclusions:**

Dependence and poor compliance may contribute to withdrawal symptoms with long-term BZD use. Providing guidance to ensure proper compliance is thought to be the best way to mitigate withdrawal symptoms.

**Trial registration:**

UMIN000024462 (18/10/2016).

## Background

Benzodiazepines and benzodiazepine receptor agonists (BZDs) including Z-drugs are widely used for the treatment of insomnia. The possibility of causing tolerance and dependence has been emphasized as a negative effect of long-term BZD use. A study associated with the United States National Health and Nutrition Examination Survey (NHANES) found that a growing proportion of patients received long-term prescriptions of BZDs, with about 3% receiving a prescription for 6 months or longer [1]. In Japan, it was also reported, based on insurance billing records, that 12% of individuals taking hypnotics or anti-anxiety drugs received a prescription for 1 year or longer [2]. Long-term BZD use can cause a wide range of physical and psychiatric withdrawal symptoms, especially with abrupt cessation or dose reduction of the drugs [3].

Although long-term BZD use may lead to increasing individuals’ need for higher doses, BZD dependence and its associated withdrawal symptoms, including seizures, hallucinations [3, 4], did not occur in randomized clinical trials with the continuous use of BZDs at common clinical doses for 6 months or 1 year [5, 6]. In these studies, patients were strictly instructed to take their allocated treatment regularly, which, however, differ widely from daily clinical practice, where the medication administration is left mainly to patients. Therefore, the extent to which withdrawal symptoms occur in patients taking BZDs for a long time in clinical settings would be different from that in the strictly regulated clinical trials. Therefore, the symptomatic features and risk factors for the occurrence of withdrawal symptoms associated with common clinical doses are poorly understood. Of even greater concern is the lack of research on the relationship between daytime withdrawal symptoms and the worsening of insomnia often occurring in patients who discontinue a hypnotic medication at a common clinical dose [7–10].

With the goal of addressing these issues, a multi-center, open-label study was conducted to observe withdrawal symptoms in patients with chronic insomnia disorder after sudden discontinuation of 24-week treatment with BZD at a common clinical dose (2 mg of eszopiclone at bedtime).

## Methods

This study was conducted in 18 medical institutions in Japan from June 2016 to July 2018. The study protocol and all amendments were approved by the ethics committee of the Institute of Neuropsychiatry. The study was also conducted in compliance with the Declaration of Helsinki and “Ethical Guidelines for Medical and Health Research Involving Human Subjects.”

Patients 20 to 64 years of age who were diagnosed as chronic insomnia disorder based on the criteria in International Classification of Sleep Disorders, Third Edition (ICSD-3) [11] were considered eligible if they met none of the following exclusion criteria: use of a hypnotic within 2 weeks of enrollment; risk of suicide; diagnosis or history of manic episodes, post-traumatic stress disorder, alcohol or drug dependence or abuse, anorexia nervosa, bulimia nervosa, or antisocial personality disorder; drug-induced insomnia; another sleep disorder (e.g., circadian rhythm disorder, restless legs syndrome, periodic limb movement disorder, sleep apnea syndrome); physical symptoms that possibly impair sleep, such as pain, fever, diarrhea, frequent urination, or cough; or a suspected organic psychiatric disorder. These exclusion criteria were set in order to minimize the influence of the factors aforementioned. All subjects took the study medication after being informed of the purpose and procedures of the study and giving written, informed consent.

As noted earlier, the subjects took 2 mg of eszopiclone every night for 24 weeks, and medication was then discontinued during a 2-week follow-up period. At the end of the follow-up period (Week 26), the subjects rated the withdrawal symptoms they experienced during the follow-up period using the Benzodiazepine Hypnotics Withdrawal Symptom Scale (BHWSS) [12]. This instrument contains 10 items (Dizziness; Feeling faint; Feeling sick; Unable to control your movement; Loss of memory; Feeling fatigue; Heart pounding [palpitations]; Feeling full-headed or headache; Feeling anxious, nervous, or jittery; and Feeling weak), each of which is assessed on a 3-grade Likert scale of 0 for no, 1 for yes-moderate, and 2 for yes-severe. We interpreted a BHWSS score of 7 points or higher to indicate clinically relevant withdrawal symptoms, according to the previous study that found 6.5 points of the BHWSS as a cutoff value for detecting at least 2 points on the “withdrawal” subscale of the Benzodiazepine Dependence Self-Report Questionnaire short version; Bendep-SRQ SV [13]. The Insomnia Severity Index (ISI) [14] was used to rate insomnia severity at baseline, during the treatment period (Week 4), at the end of the treatment period (Week 24), and at the end of the follow-up period (Week 26). To determine subject patients’ dependence and compliance during the treatment period, the Bendep-SRQ SV subscales of “problematic use”, “preoccupation”, and “lack of compliance” were assessed at the end of the treatment period (Week 24) and sum of the subscale scores was calculated (hereafter referred to Bendep-PPL score). Each of the subscales contains 5 items that are assessed on a 5-grade scale. The responses were dichotomized by taking scores of 2 or less to be 0 and scores of 3 or greater to be 1 [15].

Baseline clinical descriptive variables including age, sex, body mass index, length of insomnia, and previous history of hypnotics use were compared between the completed patients and the discontinued patients to confirm their homogeneity using Student’s *t*-test for continuous variables and Fisher’s exact test for categorical variables. The ISI score at baseline was compared to scores at Week 4, 24, or 26 using Student’s *t*-test, and those at Weeks 24 and 26 were compared using a paired *t*-test. In addition, the change in ISI scores during the follow-up period (i.e., from Week 24 to Week 26) was compared between the subjects with and without withdrawal symptoms using Student’s *t*-test to characterize the relationship of withdrawal symptoms to post-discontinuation changes in insomnia symptoms. To determine factors related to the increase in the BHWSS score, multiple forward-backward stepwise linear regression analysis was performed to evaluate the associations of the score to demographic measures (age, sex, and length of insomnia), ISI scores at Week 24, and Bendep-PPL score. Univariate and multivariable forward-backward stepwise logistic regression analyses were also performed to evaluate the associations between the presence of clinically relevant withdrawal symptoms and the same covariates described above. For this analysis, significance was examined using the Wald Chi-squared test. The significance level was set at 0.05, but for inclusion of a positive predictor in multiple stepwise regression analysis, the significance level was set at 0.15. All statistical analyses were conducted using SAS version 9.4 (SAS Institute Inc., Cary, NC, USA).

## Results

Of the 123 outpatients who consented to participate in this study, all were eligible and started receiving treatment. Of them, 77 completed the 24-week treatment, but one case failed to visit a clinic at Week 26. Thus, 47 patients discontinued the study before Week 26. Of the completed cases, 19, 7, and 2 patients had diagnoses of depressive disorders, anxiety disorders, and adjustment disorders, respectively. The major reasons for discontinuation were missed visit (*n* = 30) and withdrawal of consent (*n* = 9). Demographics and baseline features related to insomnia are shown in Table 1. Sex was the only variable with significant difference (*p* = 0.0434) between the completed and the discontinued cases.

**Table 1:** Patients’ demographic and baseline characteristics

		Completed cases (*n* = 76)	Discontinued cases (*n* = 47)
Age (y)	Mean (SD)	41.7 (12.2)	39.6 (10.9)
Sex, male	n (%)	34 (44.7%)	30 (63.8%)
Body mass index (kg/m^2^)	Mean (SD)	22.89 (4.82)	22.85 (4.87)
History of insomnia (y)	Mean (SD)	2.50 (2.98)	2.63 (4.01)
History of hypnotics use^a^	n (%)	11 (14.5%)	5 (10.6%)

*Abbreviation*: *SD* Standard deviation

^a^In a year before the date of consent

Eszopiclone treatment significantly decreased insomnia severity of the subject patients. The ISI score was 17.0 ± 3.9 at baseline (mean ± standard deviation [SD], *n* = 112), and it decreased by − 7.4 at Week 4 (effect size = − 1.4, *n* = 110, *p* < 0.001) and by − 9.5 at Week 24 (effect size = − 1.7, *n* = 76, *p* < 0.001). The beneficial effect was maintained after the 2-week follow-up period (− 8.2 at Week 26; effect size = − 1.6, *n* = 76, *p* < 0.001). However, the ISI score at Week 26 increased slightly, but significantly, from Week 24 (+ 1.4, effect size = + 0.4, *n* = 76, *p* = 0.003).

**Fig. 1:**
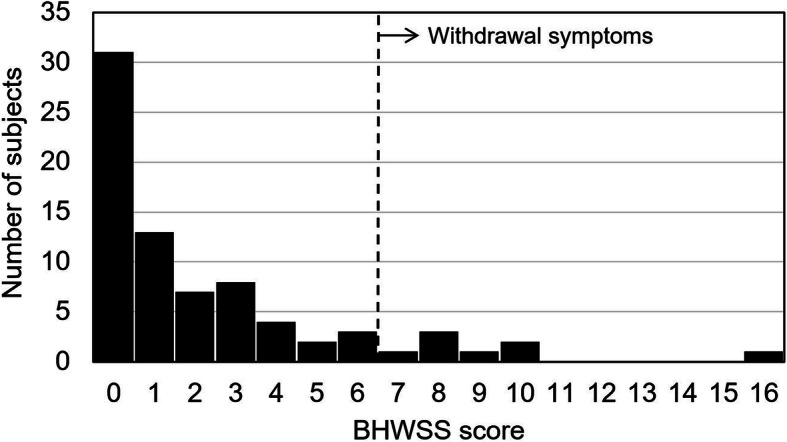
Distribution of BHWSS scores among the subjects (*n* = 76) who had clinically relevant withdrawal symptoms. BHWSS scores of 7 and greater are considered to indicate withdrawal symptoms

**Fig. 2:**
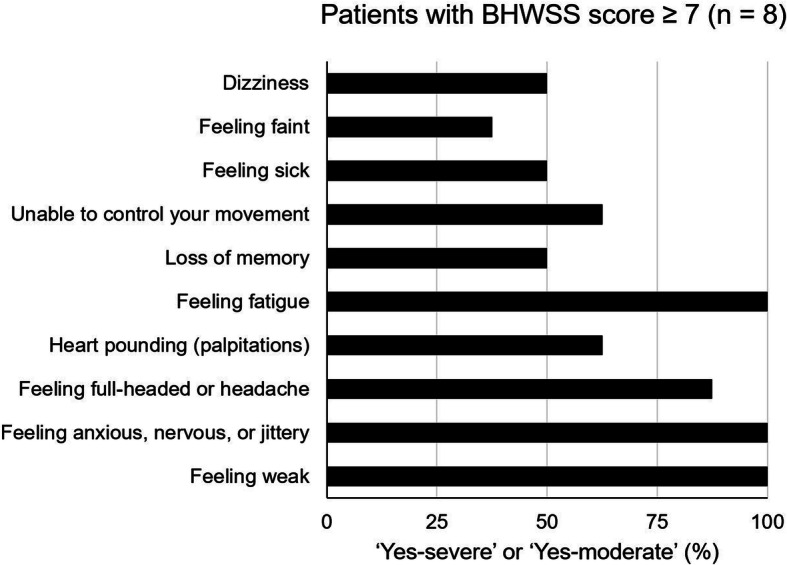
The positivity rates for the respective BHWSS items in the withdrawal symptom-positive population (*n* = 8)

The median self-evaluated BHWSS score at the end of the follow-up period was 1 (interquartile range, 0–3). Eight subjects (10.5%) had a clinically relevant withdrawal symptom (i.e., BHWSS scores ≥7); however, the BHWSS score of all but 1 of these subjects was 10 or less (Fig. 1). The most common complaints of the patients with a clinically relevant withdrawal symptom were fatigue (100%, 8/8), weakness (100%, 8/8), and anxious, nervousness, or jitteriness (100%, 8/8) (Fig. 2).

Multiple stepwise linear regression analysis showed a significant correlation between Bendep-PPL scores and BHWSS scores (B = 0.949, standard error = 0.154, *p* < 0.001), with no significant correlations with other variables (B = 0.052, standard error = 0.472, *p* = 0.913 for intercept). This indicated that dependence and medication compliance were associated with severity of withdrawal symptoms; however, as the BHWSS scores were not normally distributed, we further performed logistic regression analyses to identify factor(s) that predict clinically relevant withdrawal symptoms (Table 2). Univariate logistic regression analysis revealed that the Week 24 ISI score (odds ratio [OR], 1.169; 95% confidence interval [95% CI], 1.012–1.350; *p* = 0.034) and the Bendep-PPL score (OR, 1.664; 95% CI, 1.113–2.489; *p* = 0.013) were associated with the presence of withdrawal symptoms. On multiple logistic regression analysis, only the Bendep-PPL score was statistically significant (OR, 1.650; 95% CI, 1.105–2.464; *p* = 0.014).

**Table 2:** Univariate and multivariable logistic regression analyses of risk factors for withdrawal symptoms

	Univariate				Multivariable			
	OR	95%CI	χ^2^ value	*p*-value	OR	95%CI	χ^2^ value	*p*-value
ISI score at Week 24	1.169	1.012, 1.350	4.493	0.034				
Age (per 10 years)	0.778	0.416, 1.457	0.615	0.433				
Sex (female)	2.743	0.516, 14.58	1.401	0.237				
History of insomnia (per 1 year)	0.895	0.654, 1.227	0.474	0.491				
Bendep-PPL score	1.664	1.113, 2.489	6.148	0.013	1.650	1.105, 2.464	5.996	0.014

*Abbreviations*: *95%CI* 95% confidence interval, *ISI* Insomnia severity index, *OR* Odds ratio, *PPL* “problematic use”, “preoccupation”, and “lack of compliance”

The change in ISI scores during the follow-up period (Week 26 score minus Week 24 score) did not differ significantly between the subjects with and without clinically relevant withdrawal symptoms (mean ± SD: with withdrawal symptoms, 3.0 ± 6.0; without withdrawal symptoms, 1.3 ± 3.7; *p* = 0.245).

## Discussion

In the present study, 10.5% of the patients who abruptly discontinued treatment with a clinical dose of eszopiclone reported a clinically relevant withdrawal symptom, and hypnotic dependence and compliance were significantly correlated with symptom occurrence. A slight worsening in insomnia symptoms following discontinuation was not associated with the presence of withdrawal symptoms occurring mainly in the daytime.

As in the previously reported 6-month clinical trial with treatment discontinuation [5], no serious withdrawal symptoms such as seizures or hallucinations occurred after BZD discontinuation in the present study. The incidence of withdrawal symptoms in the present study, however, exceeded those in previous clinical trials, in which 10 to 15% of subjects experienced post-discontinuation adverse events, most of which were unlikely related to the BZD discontinuation [5, 6]. A higher incidence in the present study may suggest that the BHWSS better detects even mild withdrawal symptoms. The present result also showed that BZD hypnotic users may experience withdrawal symptoms more frequently than currently thought.

Feeling weak, feeling anxious, nervous, or jittery, and feeling fatigue were the three most frequent symptoms of hypnotic withdrawal in the present study. These symptoms should be considered in light of the possibility that the worsening of insomnia following discontinuation may have affected daytime function. Given, however, that they occur relatively commonly after the discontinuation of long-term BZD use [3], and that the worsening of post-discontinuation insomnia symptoms in the present study did not differ significantly between the subjects with and without withdrawal symptoms, these withdrawal symptoms likely occurred independently of insomnia worsening after discontinuation. Conversely, the discordance seen between withdrawal symptoms and insomnia symptoms may suggest that the post-discontinuation worsening in insomnia symptoms in the present study was not rebound insomnia (which is thought to occur in parallel with withdrawal symptoms due to dependency on BZD hypnotics), but rather occurred as the manifestation of latent and residual insomnia symptoms after discontinuation of hypnotics [16].

A notable finding of the present study was that the high Bendep-PPL score, which suggest that dependence and improper compliance on the study medication, was significantly associated with the occurrence of withdrawal symptoms. Reportedly, the formation of BZD dependence and the occurrence of resultant withdrawal symptoms are closely associated with the dose and duration of use of BZDs [3]. The present study, however, used a fixed dose and a fixed treatment period of 24 weeks, which may contribute to the clarification of the relevance of patient dependence and compliance to the development of withdrawal symptoms caused by BZD hypnotics. In other words, dependence and improper compliance resulting from the patient-guided medication regimens used in this study may partially explain why the incidence of withdrawal symptoms was higher than in previous studies [5, 6].

Several limitations to this study must be acknowledged. First, the lack of a placebo group made it difficult to determine whether withdrawal symptoms were attributable to the sudden loss of the pharmacological action of the drug or were psychological reactions of the subjects. Second, the BHWSS was not administered before the discontinuation of treatment. This left it unclear how the symptoms identified with the BHWSS evolved at treatment discontinuation, and one cannot completely rule out the possibility that the symptoms were ancillary to the chronic insomnia disorder. Third, the majority of variables in this study were collected by self-reported questionnaires. Although the reliability of BHWSS has been established in a previous study [12], a further study using objective assessments including thorough clinical interviews and physiological measures would be necessary to confirm the result of the present study. And fourth, because a considerable portion of participants had anxiety/depressive symptomatology in this study (35.4% of those who completed the 24-week treatment), it is possible that the association between Bendep-PPL with BHWSS were confounded by active anxiety/depressive symptoms of participants and that residual anxiety/depressive symptoms were detected as withdrawal symptoms by BHWSS.

## Conclusions

The present study showed that a certain number of insomnia patients receiving a common clinical dose of BZD hypnotics may develop withdrawal symptoms after treatment discontinuation, irrespective of an aggravation of insomnia symptoms, and that dependence and improper compliance on medication may contribute to the development of withdrawal symptoms. A future prospective interventional study with a placebo-controlled design would be necessary to confirm the results of the present study and to determine whether improving treatment compliance helps mitigate BZD hypnotic withdrawal symptoms.

## Data Availability

The datasets used and/or analyzed during the current study are available from the corresponding author on reasonable request.

## Abbreviations

Bendep-SRQ SV: Benzodiazepine Dependence Self-Report Questionnaire short version
BHWSS: Benzodiazepine Hypnotics Withdrawal Symptom Scale
BZD: Benzodiazepine receptor agonist
CI: Confidence interval
ICSD-3: International Classification of Sleep Disorders, Third Edition
ISI: Insomnia Severity Index
NHANES: United States National Health and Nutrition Examination Survey
OR: Odds ratio
SD: Standard deviation
